# Dose-Dependent Effects of Conjugated Linoleic Acid on Hepatic Anti-Inflammatory Activity in Mice

**DOI:** 10.3390/biology15211992

**Published:** 2026-11-04

**Authors:** Wenqi Liang, Hang Zhang, Zheming Ren, Minghui Liu

**Affiliations:** College of Animal Science and Technology, Inner Mongolia Minzu University, Tongliao 028000, China; 15947773597@163.com (W.L.); 15734868118@163.com (Z.R.); 18419336807@163.com (M.L.)

**Keywords:** conjugated linoleic acid, lipopolysaccharide, acute liver injury, inflammation, oxidative stress, *PPARγ*

## Abstract

Conjugated linoleic acid (CLA) exerts anti-inflammatory and antioxidant bioactivities, yet its optimal hepatic anti-inflammatory dosage under lipopolysaccharide (LPS) stimulation remains unclear. This study aimed to screen the effective dietary CLA dose against LPS-triggered liver inflammation in mice. Twenty-four male mice were divided into four groups (*n* = 6): normal diet (ND), ND+0.5% CLA, ND+1% CLA and ND+2% CLA. After 49 days of feeding, all mice received LPS injection and were sacrificed 24 h later. Serum and liver tissues were collected to detect pro-inflammatory cytokines (*TNF-α*, *IL-1β*, *IL-6*), the oxidative stress marker malondialdehyde (MDA), antioxidant indices (superoxide dismutase, SOD and total antioxidant capacity, TAC), hepatic inflammatory gene mRNA and liver histopathology. The 1% CLA group exhibited lower serum and hepatic pro-inflammatory cytokines (*TNF-α*, *IL-1β*, *IL-6*) and the oxidative stress marker MDA, higher SOD and TAC (*p* < 0.01), and reduced hepatic inflammatory infiltration. All CLA treatments downregulated hepatic CXCL1 mRNA and upregulated peroxisome proliferator-activated receptor γ (*PPARγ*) mRNA (*p* < 0.05). In conclusion, 1% dietary CLA elicited optimal anti-inflammatory effects in this acute LPS-induced liver inflammation model, while 1% and 2% CLA showed comparable antioxidant effects. This study was a short-term acute inflammation mouse model. The tissue distribution and lipid metabolic profile of CLA were not examined, and the CLA mixed isomers were used. Moreover, the *PPARγ* pathway was inferred based solely on mRNA levels, and further in-depth verification is still needed.

## Simple Summary

Conjugated linoleic acid is a natural fatty acid found in dairy products and beef. This study asked whether it can protect the liver from sudden inflammation, and which amount works best. Mice were fed for seven weeks with a normal diet or diets containing different amounts of this fatty acid. They were then given a bacterial substance that causes liver inflammation. Signs of inflammation and cell damage were measured. The results showed that the one percent dose was best at reducing liver inflammation. Both the one percent and two percent doses reduced cell damage equally. Unexpectedly, the two percent dose gave no extra benefit and even showed signs of possibly increasing some inflammatory signals. The study concludes that a one percent dose may be the best choice for preventing acute liver inflammation. This work is valuable because it suggests a safe and effective dietary approach that could help prevent liver damage. However, more research is needed before these findings can be applied to people.

## 1. Introduction

The liver is a crucial organ for detoxification and metabolism, responsible for processing metabolic waste, external substances, and drugs, making it susceptible to damage [1]. Acute liver inflammation can rapidly cause hepatocyte damage and extensive infiltration of inflammatory cells. Persistent acute inflammation serves as a critical initiating factor for the development of chronic liver injury, hepatic fibrosis, and even cirrhosis and hepatocellular carcinoma [2]. Early-stage prevention and treatment of liver injury are essential. Currently, many drugs exist for these purposes; however, they typically come with toxic side effects. Some natural substances offer liver protection by scavenging free radicals, thereby alleviating oxidative stress and reducing cellular inflammatory damage. Numerous natural compounds have demonstrated hepatoprotective effects. Curcumin, the major bioactive compound of Curcuma longa, exerts antioxidant and anti-inflammatory activities by inhibiting *NF-κB* activation and reducing *IL-6* expression [3]. Resveratrol decreases hepatic inflammation (*IL-6* and *IL-1β*) and oxidative stress markers and improves antioxidant enzyme activities including SOD and catalase [4]. Quercetin protects against drug-induced hepatotoxicity by activating *NRF2* and blocking the *NF-κB/TLR-4* axis [5]. Compared to synthetic drugs, these natural substances generally have fewer side effects. Consequently, interest in using natural substances for liver injury prevention and treatment is growing [6]. Several synthetic compounds are currently used to reduce oxidative stress and inflammation in liver damage. N-acetylcysteine (NAC) is used for acetaminophen-induced hepatotoxicity, but may cause nausea, vomiting and anaphylactoid reactions [7]. Corticosteroids suppress inflammation but can lead to immunosuppression, osteoporosis, hyperglycemia and hypertension [8]. Pentoxifylline, a phosphodiesterase inhibitor with antioxidant and anti-inflammatory properties, may cause gastrointestinal upset and dizziness [9]. These limitations, including toxic side effects and narrow therapeutic windows, highlight the need for safer alternatives. Owing to their advantages of low toxicity and multi-target regulation of inflammation and metabolism, complementary therapies and natural bioactive compounds have become a major research focus in the field of modern chronic disease prevention and adjunctive intervention, particularly for obesity, immune disorders, and chronic inflammatory diseases [10,11].

Polyunsaturated fatty acids (PUFAs) are important bioactive molecules that regulate cellular inflammatory responses and oxidative stress homeostasis through multiple mechanisms, including modulation of eicosanoid production, inhibition of *NF-κB* activation, and regulation of antioxidant enzyme expression [12,13]. As an important subtype of PUFAs, conjugated linoleic acid (CLA) has attracted considerable attention due to its significant anti-inflammatory and antioxidant activities. CLA is a mixture of positional and geometric isomers of linoleic acid (LA) with conjugated double bonds. In naturally occurring CLA, cis-9, trans-11-CLA accounts for 80–90%, whereas trans10,cis12-CLA accounts for only 3–5% [14]. Nevertheless, CLA produced by in vitro bioconversion generally contains comparable amounts of these two isomers. For this reason, many studies employ a mixture of the two isomers to explore the biological functions of CLA [15,16,17]. Accumulating evidence demonstrates that CLA can modulate inflammatory responses and oxidative stress in vivo through multiple pathways, including *PPARγ*/*NF-κB*, *TLR4*, *COX-2* and *Nrf2* signaling [18]. However, the dose-dependent effects of CLA in acute LPS-induced liver inflammation, especially the optimal dietary dose, remain incompletely defined.

CLA plays a significant protective role in immune and inflammatory responses [19,20]. Dietary CLA supplementation has been shown to suppress colon inflammation in pigs with bacteria-induced colitis [21]. It also reduces inappropriate colonic and uncontrolled intestinal mucosal inflammation. Current research on CLA has largely focused on its chronic metabolic effects, such as improving obesity-related lipid metabolism, modulating intestinal immune inflammation, and alleviating hepatic steatosis [22]. Although several studies have reported that CLA or CLA-enriched diets attenuate LPS-induced inflammation, including early intervention studies showing that dietary CLA pretreatment reduces LPS-induced inflammation [23,24], few studies have systematically compared graded dietary CLA doses in an acute LPS-induced liver inflammation model. The optimal dietary dose therefore remains unclear.

The present study was designed to investigate the preventive effect of dietary CLA supplementation against LPS-induced acute hepatic inflammation in healthy normal-diet-fed mice. Specifically, we aimed to screen the optimal dietary CLA dosage by detecting serum and hepatic pro-inflammatory cytokines, antioxidant indicators, hepatic inflammation-related gene mRNA expression and liver histopathological changes. The findings will provide experimental evidence for the nutritional preventive application of CLA against acute endotoxin-triggered liver injury. These efforts will ultimately refine the theoretical positioning of CLA within the “dietary fatty acid-inflammatory regulatory network” and provide more comprehensive scientific support for its application in dietary intervention and drug development for liver inflammation-related diseases. Future work will further dissect the molecular mechanisms underlying CLA-mediated regulation of liver inflammation, clarify the functional differences and synergistic or antagonistic effects of its two major isomers, and extend the investigation to additional liver inflammation models to validate both its anti-inflammatory efficacy and its potential pro-oxidant/pro-inflammatory effects.

## 2. Materials and Methods

### 2.1. Animals and Treatments

Twenty-four male C57BL/6J mice (body weight: 37.79 ± 2.17 g) were obtained from the Academy of Military Medical Sciences in Beijing, China [approval number: SCXK (Jing) 2019-0010]. CLA was supplied by Aohai Biological Co., Ltd., Qingdao, China, with a purity of 80.4%. The composition was 38.5% cis-9, trans-11-CLA, 38.3% trans10, cis12-CLA, and 3.6% other CLA isomers. The remaining components were 10.8% oleic acid, 2.8% stearic acid, 3.1% palmitic acid, 1.5% LA, and 1.4% unidentified substances. The mice feed was procured from SPF Biotechnology Co., Ltd., Beijing, China. The study protocol was reviewed and approved by the Animal Care and Ethics Committee of Inner Mongolia Minzu University (No. NMD-DW-2024-11-15). The mice were housed in laboratory cages under controlled conditions (22 ± 2 °C and 60–70% humidity) with a 12-h light/dark cycle. Food and water were provided ad libitum. Following a 7-day acclimatization period, the mice were randomly assigned to four groups, (*n* = 6 per group): a normal diet (ND) group, a normal diet plus 0.5% CLA (CLA-0.5) group, a normal diet plus 1% CLA (CLA-1) group, and a normal diet plus 2% CLA (CLA-2) group. The CLA doses are expressed as mass-to-mass percentage of CLA raw material (*w*/*w*; g of CLA raw material per 100 g basal diet), not solution volume. Given that the CLA raw material had a purity of 80.4%, the corresponding pure CLA equivalents were approximately 0.402%, 0.804% and 1.608% for the 0.5%, 1% and 2% groups, respectively. These doses were selected based on previous rodent studies [22] and human equivalent dose (HED) calculations. Briefly, based on the average daily feed intake (4.5 g/day) and mean body weight (37.79 g) of mice in this study, the approximate daily CLA intake was 22.5 mg/day for the 0.5% group, 45 mg/day for the 1% group, and 90 mg/day for the 2% group. The corresponding body-weight-normalized doses were approximately 0.60, 1.19, and 2.38 g/kg·d, respectively. Using the BSA normalization method (Meeh–Rubner formula: BSA(m^2^) = k × (body weight g)^2/3^ × 10^−6^, K_mouse_ = 9.1, K_human_ = 10.1), the HED for a 60-kg adult corresponding to 1% dietary CLA in mice was approximately 4.8 g/day after correction for the average mammalian dietary CLA bioavailability of 65.6%. This value falls within the clinically recommended daily supplementation range (3–6 g CLA/day) for healthy adults and is lower than the recommended daily linoleic acid intake (14–16 g/day), without risk of metabolic overload. CLA raw material was thoroughly blended into the basal diet to produce experimental pelleted feed. No gavage or injection dosing based on individual body weight was applied.

After 7 weeks (49 days) of dietary pretreatment with or without CLA, all four groups received an identical intraperitoneal LPS challenge to establish an acute liver inflammation model; no saline-injected non-inflamed control group was included. Therefore, the ND group served as the LPS-inflamed control, and all comparisons reflect the effects of dietary CLA on LPS-induced acute liver inflammation. Each mouse received an intraperitoneal injection of 50 µL of 0.2 mg/mL LPS (L2630, Sigma, St. Louis, MO, USA). At 24 h after LPS injection, mice were anesthetized with 3% isoflurane (RWD Life Science, Shenzhen, China). Blood was then collected via retro-orbital bleeding, and mice were sacrificed by cervical dislocation while still under anesthesia. The full experimental workflow is visualized in Supplementary Figure S1.

### 2.2. Blood and Tissue Collection for Biochemical Analysis

Blood (0.4–0.6 mL per mouse) was collected via retro-orbital bleeding (orbital sinus) into a 1.5 mL centrifuge tube (Sorfa, Hangzhou, China) without anticoagulant. The blood samples were left at room temperature and then centrifuged at 4 °C at 3000 rpm for 15 min. Subsequently, the supernatant serum was separated and stored in polypropylene microtubes (Sorfa, Hangzhou, China) at −80 °C until analysis of pro-inflammatory cytokines (*TNF-α*, *IL-1β*, *IL-6*), the oxidative stress marker MDA, SOD activity, and TAC. All assays were performed using commercial kits according to the manufacturer’s instructions. MDA content was expressed as nmol/mL, SOD activity as U/mL, and TAC as U/mL or mmol Trolox equivalent/L. The liver was harvested, weighed, and stored at −80 °C for subsequent analysis of TNF-α, IL-1β and IL-6 protein in the supernatants, which were measured using ELISA kits (Shanghai Enzyme-linked Biotechnology Co., Ltd, Shanghai, China) according to the manufacturer’s instructions and detected by an Infinite^®^ 200 PRO multifunctional microplate reader (Tecan Trading AG, Männedorf, Switzerland).

Liver tissues were weighed and homogenized in cold PBS (1:9, *w*/*v*; final concentration 0.1 g tissue/mL) using a tissue homogenizer(Shanghai Jingxin Industrial Development Co., Ltd., Shanghai, China). The homogenates were centrifuged at 12,000× *g* for 15 min at 4 °C, and the supernatants were collected. The concentrations of *TNF-α*, *IL-1β* and *IL-6* in the supernatants were measured using ELISA kits (Shanghai Enzyme-linked Biotechnology Co., Ltd., Shanghai, China) according to the manufacturer’s instructions. Because protein quantification was not performed, the hepatic cytokine concentrations were normalized to tissue weight according to the homogenization ratio. The final hepatic cytokine levels were calculated as hepatic cytokine (ng/g tissue) = supernatant cytokine (ng/L) ÷ 100 and expressed as ng/g liver tissue.

### 2.3. Histopathological Study

The histopathological evaluation method used was that of Bancroft and Gamble (2008) [25]. The liver tissue biopsies were fixed in 10% neutral-buffered formalin (Sigma, St. Louis, MO, USA). The specimens were then cleaned, dehydrated using alcohol, cleared in xylene, and embedded in paraffin. Sections measuring 5 μm were cut and stained with hematoxylin and eosin (H&E) for examination. We conducted a histopathological examination of the specimens under a light microscope (Eclipse Ci-s; Nikon, Tokyo, Japan) to analyze the characteristics of the samples. Liver sections were examined under a light microscope (Eclipse Ci-s; Nikon, Japan) by two independent observers blinded to group allocation. In this short-term acute LPS model, hepatic lesions were predominantly mild focal inflammatory cell infiltration, without overt hepatocyte necrosis or sinusoidal congestion. Therefore, histopathological changes were assessed descriptively, and a formal semi-quantitative scoring system was not applied. The identities of the groups were concealed during evaluation. A standardized semi-quantitative scoring system was not applied, as overt hepatocyte necrosis and sinusoidal congestion—features typically subjected to grading—were not apparent in this short-term acute model.

### 2.4. Quantitative Real-Time Polymerase Chain Reaction (qRT-PCR)

The mRNA abundances of TNF-α, Cxcl1 (CXCL1, functional homolog of CXCL1) and *PPARγ* in the liver were detected using qRT-PCR. Briefly, liver samples were ground into powder in a pre-cooled mortar with liquid nitrogen, and RNAiso Plus lysis reagent was then added. Total RNA was extracted using the RNAiso Plus kit (TaKaRa, 9108, Dalian, China) following the manufacturer’s instructions and stored at −80 °C. RNA purity and concentration were assessed by measuring the 260 nm/280 nm ratio with a Nucleic Acid Protein Ultramicrospectrophotometer (Pultton P100+, Sunnyvale, CA, USA). The total RNA was reverse transcribed into cDNA using the PrimeScript RT Master Mix Kit (TaKaRa, RR820A, Dalian, China). The reverse transcription reaction involved a temperature program of 37 °C for 15 min and 85 °C for 5 s. RT-qPCR was conducted on a Light Cycler 480 (Roche Holding AG, Basel, Switzerland) using a SYBR Premix Ex TaqTM II kit (TaKaRa, RR047A, Dalian, China), in accordance with the manufacturer’s instructions. The PCR conditions included an initial denaturation at 95 °C for 30 s, followed by 40 cycles of 95 °C for 30 s, 60 °C for 20 s, and 72 °C for 30 s. Glyceraldehyde-3-phosphate dehydrogenase (GAPDH) served as the internal control for mRNA expression in our experiments. The primer sequences, designed and synthesized by Shanghai Sangon Biological Engineering and Technology Service Co. Ltd., Shanghai, China, are detailed in Table 1.

### 2.5. Statistical Analysis

We analyzed at least three biological replicates from each treatment group. All data are expressed as mean ± standard error of the mean (SEM). Prior to statistical analysis, the Shapiro–Wilk normality test was used to assess the distribution of the data. All measured data were normally distributed (*p* > 0.05). One-way analysis of variance (ANOVA) was performed to determine the overall differences among groups, followed by Duncan’s multiple range test for multiple comparisons among all groups. Values with different lowercase letters (a–c) indicate significant differences at *p* < 0.05. All statistical analyses were conducted using SAS 9.0 software. For all gene transcript abundances, data were obtained as cycle threshold (CT) values, and 2-∆∆Ct values were calculated. A *p* value less than 0.05 was considered statistically significant.

## 3. Results

All mice received an intraperitoneal LPS challenge and were sacrificed 24 h later. Serum and liver samples were collected for the following analyses.

### 3.1. Effect of Different CLA Levels on Serum Inflammatory Factors

As shown in Figure 1, serum *IL-1β* and *IL-6* were significantly reduced in the CLA-0.5 and CLA-1 groups (*p* < 0.05). Serum TNF-α was significantly reduced in the CLA-0.5 and CLA-1 groups, whereas the CLA-2 group did not differ significantly from the ND group. The data indicate that dietary supplementation with 1% CLA was most effective in reducing serum pro-inflammatory cytokines in LPS-challenged mice. In the CLA-1 group, *IL-1β*, *IL-6*, and *TNF-α* decreased by 24.95%, 33.68%, and 35.64%, respectively.

### 3.2. Effect of CLA on Hepatic Levels of Inflammatory Factors

The CLA-1 group significantly reduced pro-inflammatory cytokines (*TNF-α*, *IL-1β*, and *IL-6*) in the liver of mice (*p* < 0.01) (Figure 2). In contrast, hepatic IL-1β and TNF-α levels in the CLA-2 group were not significantly different from the ND group and did not show further reduction compared with the CLA-1 group. The results indicate that adding 1% CLA to the diet most effectively decreased inflammation induced by LPS in the livers of mice. The CLA-1 group showed decreases in *IL-1β*, *IL-6*, and *TNF-α* levels of 63.52%, 50.88%, and 68.17%, respectively.

### 3.3. Effect of CLA on Hepatic mRNA Expression of Inflammatory Factors

As depicted in Figure 3, the gene expression of TNF-α in the CLA-1 group was significantly lower than in the ND group (*p* < 0.05). Mice fed a diet with CLA exhibited decreased CXCL1 mRNA expression and a considerable increase in the relative gene expression of *PPARγ* (*p* < 0.05).

### 3.4. Effects of Different CLA Levels on Serum Oxidative Stress and Antioxidant Indices in LPS-Challenged Mice

Compared with the ND group, mice fed CLA-supplemented diets showed significantly reduced serum MDA content and increased serum SOD activity and TAC (*p* < 0.01) (Figure 4). The data revealed that dietary supplementation with CLA enhanced serum antioxidant capacity in LPS-challenged mice. Both 1% and 2% CLA significantly increased SOD activity and TAC compared with the ND group, with no significant difference between the two doses. The CLA-1 group demonstrated increases in SOD activity and TAC of 32.56% and 20.03%, respectively.

### 3.5. Histopathological Examination

As shown in Figure 5, the microscopic examination of liver sections revealed that in the ND group, the liver cells (a) had a darker color, and there were more inflammatory cells around the central vein (b) (Figure 5A); in the CLA-0.5 group and the CLA-2 group, the liver cells (a) had normal morphology, the inflammatory cells around the central vein (b) were fewer, and there was no significant difference in the degree of inflammation. The hepatic sinusoidal cells (c) showed mild activation but intact structure (Figure 5B,D), and their infiltration degree was lower than that of the ND group. It is noteworthy that in the CLA-1 group, the liver cells (a) had normal morphology, the inflammatory cells around the central vein (b) were the fewest, and the hepatic sinusoidal cells (c) had an intact structure (Figure 5C). The inflammation infiltration degree of the CLA-1 group was significantly lower than that of the other groups. These results indicate that dietary supplementation with 1% CLA exerted the most significant anti-inflammatory effect. Taken together, the elevated serum and hepatic pro-inflammatory cytokines (Figure 1 and Figure 2), up-regulated hepatic TNF-α and CXCL1 mRNA (Figure 3), as well as prominent focal inflammatory-cell infiltration around central veins observed in the ND group (Figure 5A), provide multi-dimensional biochemical, molecular and histopathological evidence confirming that LPS challenge successfully triggered acute hepatic injury in all experimental animals.

## 4. Discussion

In the present study, the serum and hepatic levels of *TNF-α*, *IL-1β* and *IL-6* in LPS-challenged mice were consistent with cytokine-mediated hepatic inflammation. Moreover, mice supplemented with the CLA isomer mixture (c9,t11-CLA and t10,c12-CLA) showed lower serum and hepatic levels of *TNF-α*, *IL-1β* and *IL-6* than the ND group, which was consistent with previous studies [26]. Moreover, a reduction in the transcription of pro-inflammatory cytokine genes was observed in the group supplemented with 1% CLA. These results indicate that the protective effect of CLA on mice against inflammation is dose-dependent. In a mouse model, dietary consumption of c9, t11-CLA has been effective in preventing Alzheimer’s disease progression by lowering amyloid β-protein accumulation and increasing levels of anti-inflammatory cytokines [27], further supporting the immunosuppressive and anti-inflammatory effects of CLA in mammalian model systems. Consistent with these observations, prior research has demonstrated that supplementation with CLA-enriched butter reduces serum TNF-α, IL-2 and IL-8 concentrations, alleviating systemic inflammation in overweight adults with subclinical inflammation [28].

Pathological sections of mouse livers can provide a more intuitive observation of inflammatory manifestations and characteristics. The major changes observed in the liver after LPS treatment were many aggregates of infiltrating macrophages surrounding the hepatocytes and central vein. An in vitro study on the anti-inflammatory effects of c9, t11-CLA and t10, c12-CLA on cultured peripheral blood mononuclear cells found that these CLA isomers suppressed the production and expression of *TNF-α* and *IL-6* [29,30]. Additionally, trans-10, cis-12 CLA significantly suppresses the production of *IL-1β* and *IL-6* and upregulates *PPARγ* expression in rumen epithelial cells under inflammatory stimulation. The protective effects of c9, t11-CLA on bovine mammary epithelial cells exposed to *Escherichia coli* (*E. coli*)-induced inflammation involve the suppression of *IL-1β*, *IL-6*, and *CXCL1* upregulation, alongside enhanced expression of anti-inflammatory cytokines [31]. Our previous study showed that cis-9,trans-11-CLA reduced *IL-6* and *CXCL1* protein levels and downregulated the mRNA expression of *IL-6*, *CXCL1*, and *IL-1β* in bovine mammary epithelial cells [32]. Results from the present study, along with findings from other research [33], suggest that reduced gene expression of pro-inflammatory cytokines could correlate positively with lowered transcription levels, contributing to a harmonized inflammatory response.

This study provides preliminary evidence that CLA upregulates *PPARγ* mRNA in the liver, suggesting a possible involvement of *PPARγ*-related signaling. However, *NF-κB* was not measured at the protein or functional level, and the causal role of *PPARγ/NF-κB* signaling in CLA-mediated hepatic protection remains to be verified by further experiments. CLA has anti-inflammatory properties, and its mechanism of action is partly mediated by the stimulation of PPARs [34]. PPARs belong to the nuclear receptor family of ligand-inducible transcription factors that regulate genes involved in adipogenesis, inflammation, lipid metabolism, mitochondrial biogenesis, and the maintenance of metabolic homeostasis [35]. *PPARγ* inhibits the production of inflammatory mediators such as *TNF-α* and *IL-6* by physically interacting with *NF-κB* [36]. CLA treatment significantly decreased the protein levels of *TNF-α*, *IL-1β* and *IL-6* measured by ELISA. In addition, *TNF-α* mRNA was downregulated, consistent with the protein data. Furthermore, it increased *PPARγ* mRNA expression and improved histological damage [37]. In an atherosclerosis model, CLA activates the *PPARγ* pathway, inhibits the expression of pro-inflammatory factors, and promotes the polarization of mononuclear macrophages towards an anti-inflammatory phenotype [38]. CLA supplementation has been shown to reduce IL-1β and *TNF-α* in the colonic tissue of mice with colitis and up-regulate *PPARγ*, thereby enhancing the intestinal inflammatory barrier [39]. Our finding, demonstrating upregulation of *PPARγ* mRNA in the liver of mice treated with CLA, aligns with studies suggesting that CLA may inhibit the transcription of pro-inflammatory cytokines through *PPARγ*-related signaling [40]. However, as the present data are limited to the transcriptional level; the causal involvement of *PPARγ* protein activation in CLA-mediated hepatic protection remains to be verified by further functional experiments.

Beyond *PPARγ* signaling, which was assessed at the mRNA level in this study, multiple other mechanisms have been proposed for CLA. Several reviews have summarized the anti-inflammatory and antioxidant actions of CLA and emphasized that its effects depend on isomer, dose and duration [41,42,43]. Specific studies have shown anti-inflammatory effects of CLA in endothelial cells [44], suppression of *NF-κB* signaling [18], anti-inflammatory effects in bovine mammary epithelial cells [40], and effects on hepatic α-tocopherol status [45]. In LPS-related models, Reynolds et al. [19] reported that a CLA-enriched diet attenuated LPS-induced inflammation partly via *PPARγ*-mediated TLR4 signaling, Li et al. [24] showed that CLA inhibited *COX-2* expression in vitro and in vivo, and Zhang et al. [32] demonstrated that linoleic acid alleviated LPS-induced acute liver injury via *Nrf2* activation. These findings indicate that CLA/LA-mediated hepatoprotection involves multiple pathways. Our study extends these observations by comparing graded CLA doses and showing a non-linear dose response, with 2% CLA providing no additional benefit and even leading to higher hepatic TNF-α and IL-1β than 1% CLA.

In our data, the 2% CLA group showed hepatic TNF-α and IL-1β levels significantly higher than the CLA-1 group and comparable to the ND group, despite receiving the highest CLA dose. This suggests a possible ceiling effect or a high-dose pro-inflammatory effect of mixed CLA, possibly related to the relatively high proportion of t10,c12-CLA in the preparation (38.3%). Therefore, 1% rather than 2% CLA was identified as the optimal dose in this acute LPS model. However, because direct pro-oxidant markers and purified isomers were not measured, this interpretation remains speculative and requires further validation.

With regard to effects of CLA on lipid peroxidation, we found a significant reduction in serum MDA levels, suggesting that CLA could act as antioxidant agents, which is in agreement with several studies about CLA [30]. CLA supplementation can prevent the increase in serum MDA levels found in mice, confirming the beneficial effect of CLA in reducing this non-enzymatic product of lipid oxidation in vivo.

CLA enhances TAC by increasing intracellular antioxidants such as glutathione and improving the expression of antioxidant enzymes, including SOD, catalase, and glutathione peroxidase [46]. Feed supplementation with CLA significantly increases SOD activity in the serum and liver, thereby enhancing the clearance of hydroxyl radicals and superoxide anions in laying hens [47]. Similarly, supplementation with CLA, which comprises 37.9% c9, t11-CLA and 39% t10, c12-CLA, reduces inflammation and oxidative stress in mice. In mice with colitis, administering a 50:50 isomer blend of c9, t11-CLA and t10, c12-CLA reduces both inflammation and oxidative stress [48]. Our study found that CLA supplementation reduces MDA levels and increases antioxidant enzymes SOD and TAC in mice serum. These outcomes align with the findings of the aforementioned studies. It is clear that CLA has a positive effect on oxidative stress, as many studies have shown that CLA has antioxidant properties in vivo models, which is consistent with our findings. Numerous studies have focused on the regulatory effects of natural bioactive substances on inflammation and metabolic homeostasis, providing an important reference for the present study. For example, natural bioactive extracts derived from plants and animals, such as bitter melon (*Momordica charantia* L.) and royal jelly, have been shown to effectively modulate inflammatory markers and improve glucolipid metabolism in humans [49,50]. Owing to their favorable biocompatibility, these natural compounds exhibit considerable potential in alleviating low-grade systemic inflammation. Compared with the aforementioned plant-derived extracts, CLA, as a natural functional fatty acid, possesses superior lipid solubility and high bioavailability, enabling it to directly target the liver and regulate inflammation-related signaling pathways. This highlights its unique advantage as a dietary functional additive in the intervention of hepatic inflammatory injury.

CLA is naturally abundant, well absorbed and has a high bioavailability, making it easy for humans to ingest and incorporate into body tissues and blood. In fact, CLA was recognized as a safe food ingredient [17], yet its safety for human consumption also warrants consideration. Some studies highlight that CLA has potential health risks, such as affecting glucose homeostasis, hepatic steatosis and milk-fat depression. Despite the concerns, these risks are generally not considered to pose significant long-term health effects. Although the human-equivalent dose calculation was provided in Materials and Methods, direct extrapolation of mouse CLA dosage to human application should be treated cautiously, considering inter-species differences, isomer composition, and individual physiological status.

This study has several limitations. First, this is a short-term acute-inflammation model induced by LPS, so only short-term hepatic regulatory effects of CLA were assessed, and its long-term impacts on chronic liver inflammation and lipid metabolic disorders remain unknown. Moreover, we did not quantify tissue enrichment of CLA isomers, hepatic fatty-acid profiles or downstream metabolites, leaving the in-vivo metabolic distribution of CLA uncharacterized, constrained by experimental objectives, duration and detection platforms. Second, all mice were subjected to LPS challenge, lacking a non-inflamed saline-injected control group; our conclusions therefore apply only to LPS-triggered acute liver injury. Third, a mixed-isomer CLA preparation was used, and the respective contribution of c9,t11-CLA and t10,c12-CLA to hepatoprotection cannot be distinguished, requiring further assays with purified isomers. Finally, the involvement of *PPARγ* was inferred merely from mRNA data. Further validation including Western blot for *PPARγ* protein and detection of its target genes (e.g., *CD36*, *adiponectin*) is needed to confirm this pathway.

Notwithstanding these limitations, the graded CLA dosages produced consistent dose-dependent responses in inflammatory cytokines, oxidative-stress markers, histopathology and key gene expression, supporting the reliability of our main findings. Future work will prolong feeding periods and analyze tissue lipid profiles to further clarify the metabolic and molecular mechanisms of CLA, laying a more solid foundation for its clinical translation as a dietary functional component against liver injury.

## 5. Limitations

Several limitations should be acknowledged. First, no saline-injected non-inflamed control group was included; therefore, the conclusions apply only to LPS-challenged conditions, and the basal effects of CLA on the liver could not be assessed. Second, hepatic cytokine levels were measured in tissue homogenate supernatants and normalized to tissue weight (ng/g liver tissue) rather than protein content, because BCA protein quantification could not be performed. In addition, the qPCR and ELISA panels were not fully matched: qPCR measured *TNF-α*, *CXCL1* and *PPARγ* mRNA, whereas ELISA measured TNF-α, IL-1β and IL-6 protein. Only *TNF-α* was measured at both mRNA and protein levels, and *IL-1β/IL-6* mRNA were not measured, which limited systematic transcript–protein comparisons. Third, histopathological evaluation was performed descriptively by two independent observers blinded to group allocation; however, a formal semi-quantitative scoring system was not applied. Fourth, the involvement of *PPARγ* was inferred only from mRNA data, and *NF-κB* protein was not measured. Direct pro-oxidant markers (e.g., ROS, GSH/GSSG, 8-OHdG) and purified CLA isomers were not assessed; thus, the potential high-dose and t10,c12-CLA-related pro-oxidant/pro-inflammatory effects cannot be excluded. These limitations should be considered when interpreting the data. Nevertheless, the main finding that 1% CLA showed the most favorable anti-inflammatory profile under LPS challenge remains supported by the consistent cytokine and histopathological results, while 1% and 2% CLA showed comparable antioxidant effects.

## 6. Conclusions

This study demonstrated that a diet supplemented with 1% CLA enhanced the activities of antioxidant enzymes and significantly reduced the levels of MDA and pro-inflammatory cytokines in both the serum and liver of LPS-challenged mice compared with the ND group. Additionally, inflammatory infiltration was markedly reduced in the CLA-1 group. In the liver of CLA-treated mice, CXCL1 mRNA expression was significantly decreased, while *PPARγ* mRNA expression was significantly increased. In conclusion, our findings demonstrate that 1% dietary CLA exerts potent anti-inflammatory activity in mouse liver. Nevertheless, inconsistent outcomes from human trials remind us that clinical translation should carefully consider CLA dosage, isomer composition and administration strategy rather than simply adopting animal-derived doses. We propose that CLA may serve as a promising nutritional intervention for hepatic inflammatory injury; further well-controlled clinical investigations are required to define optimal supplementation regimens and evaluate long-term safety before its routine therapeutic application. Future studies should extend the animal feeding duration and employ chronic inflammation models to evaluate the long-term protective effects of CLA. Furthermore, cell-based experiments and molecular omics approaches can be adopted to deeply dissect its regulatory pathways, thereby refining the theoretical framework for CLA application in the field of animal nutrition and health. In conclusion, our findings demonstrate that 1% dietary CLA exerts the most favorable anti-inflammatory activity in mouse liver under LPS challenge, while 1% and 2% CLA show comparable antioxidant effects. These conclusions are limited to the acute liver inflammation model triggered by LPS, because no saline-injected non-inflamed control group was included.

## Figures and Tables

**Figure 1:**
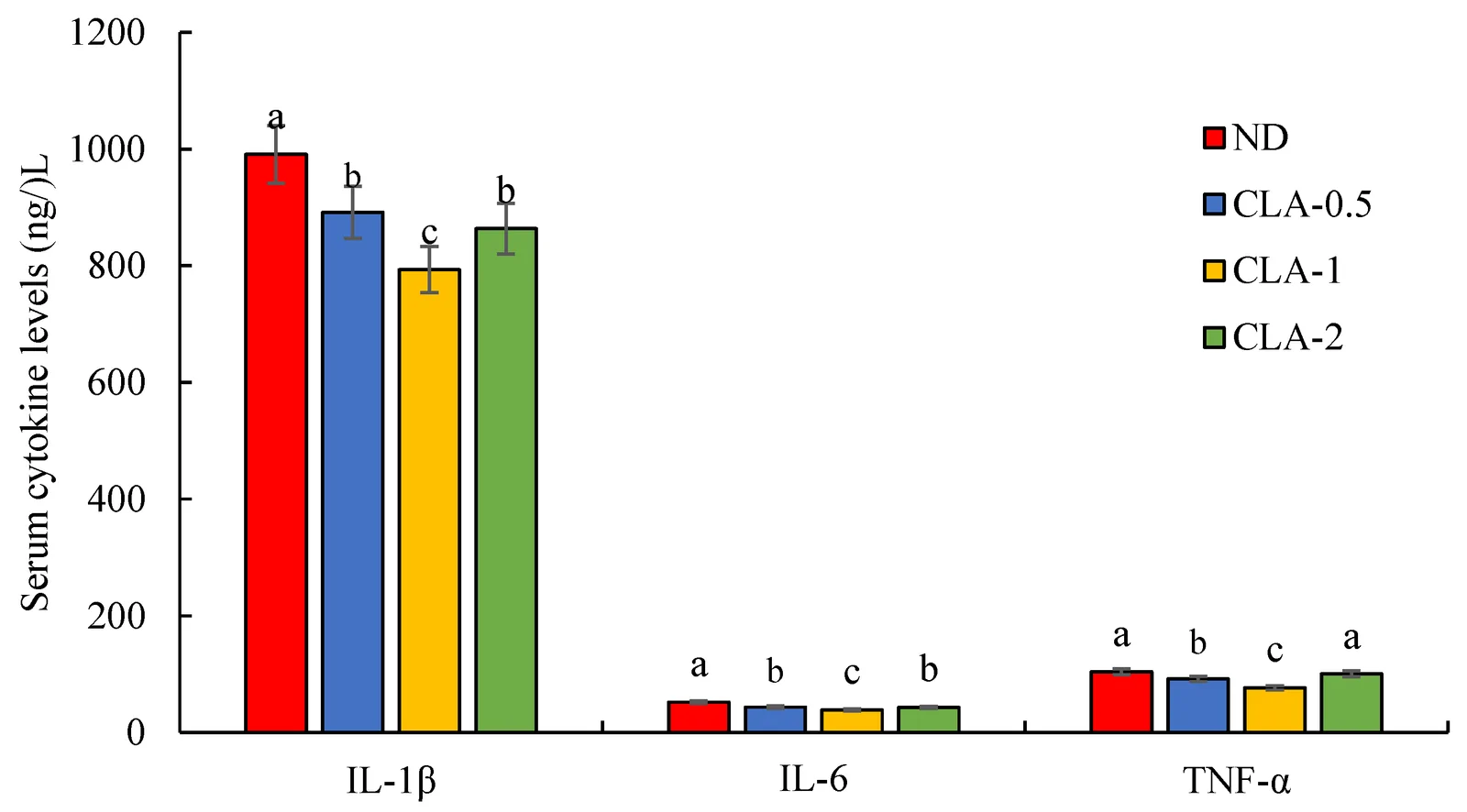
Serum levels of *IL-1β*, *IL-6*, *TNF-α* in C57BL/6J mice fed diets containing different CLA concentrations. All mice received LPS challenge, and serum samples were collected 24 h after LPS injection. Values are presented as means ± SEM (*n* = 6 per group). Values with different lowercase letters (a–c) indicate significant differences at *p* < 0.05 according to Duncan’s multiple range test.

**Figure 2:**
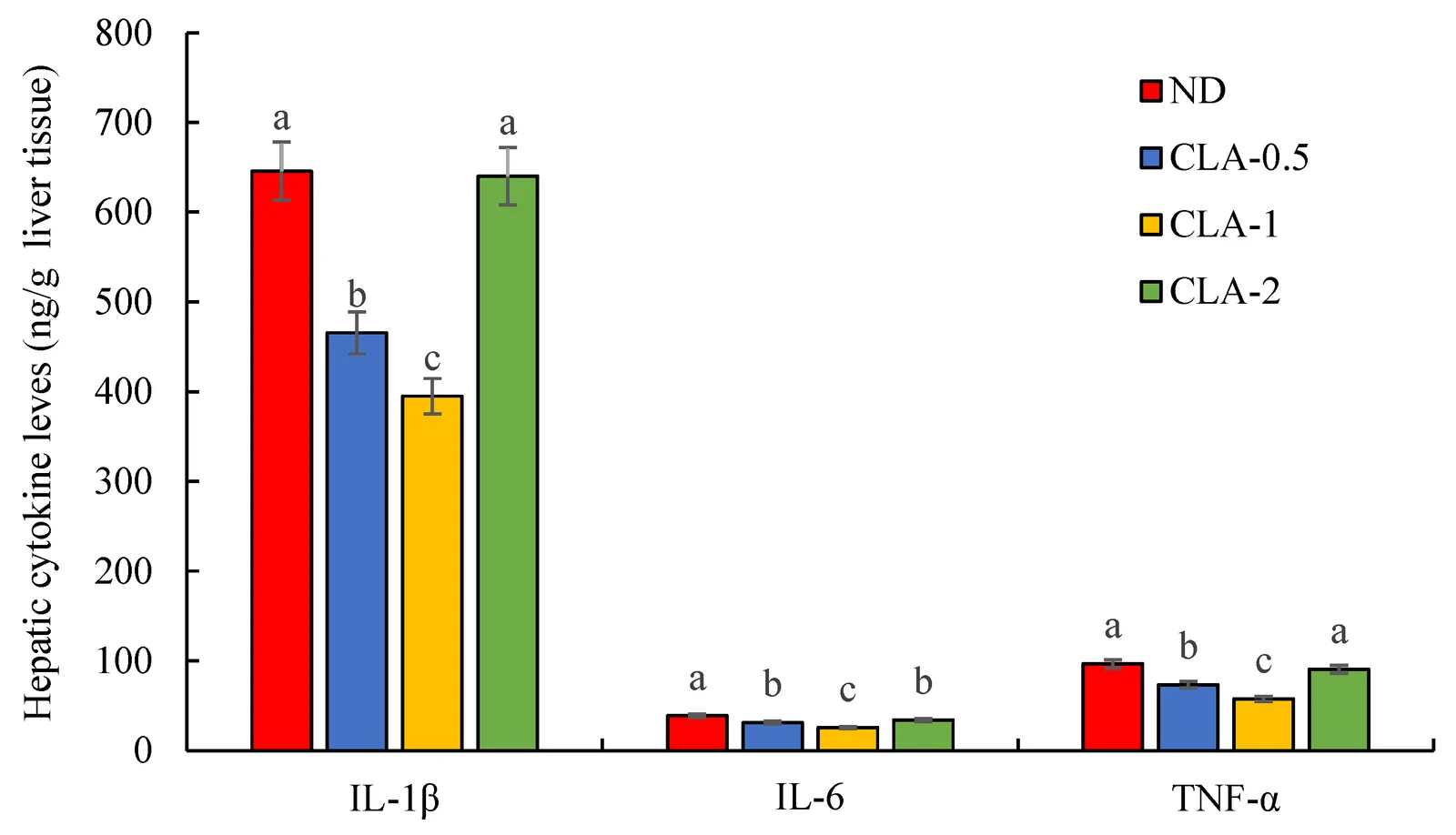
Liver levels of *IL-1β*, *IL-6*, *TNF-α* in C57BL/6J mice fed diets containing different CLA concentrations. Liver samples were collected 24 h after LPS challenge. Values are presented as means ± SEM (*n* = 6 per group). Values with different lowercase letters (a–c) indicate significant differences at *p* < 0.05 according to Duncan’s multiple range test.

**Figure 3:**
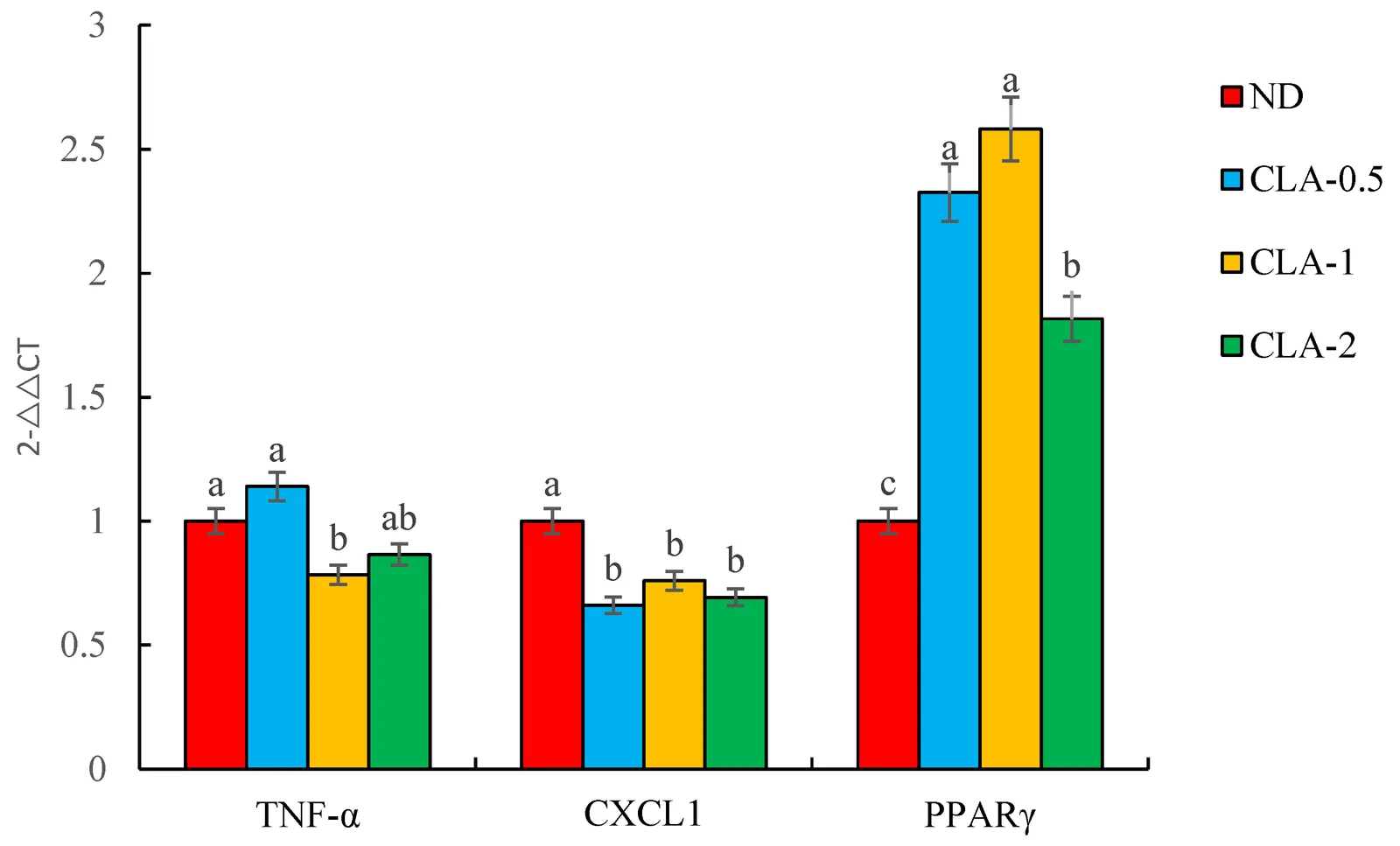
Effects of CLA on the expressions of *TNF-α*, *CXCL1* and *PPARγ* mRNAs in different groups. Liver samples were collected 24 h after LPS challenge. Values are presented as means ± SEM (*n* = 6 per group). Values with different lowercase letters (a–c) indicate significant differences at *p* < 0.05 according to Duncan’s multiple range test.

**Figure 4:**
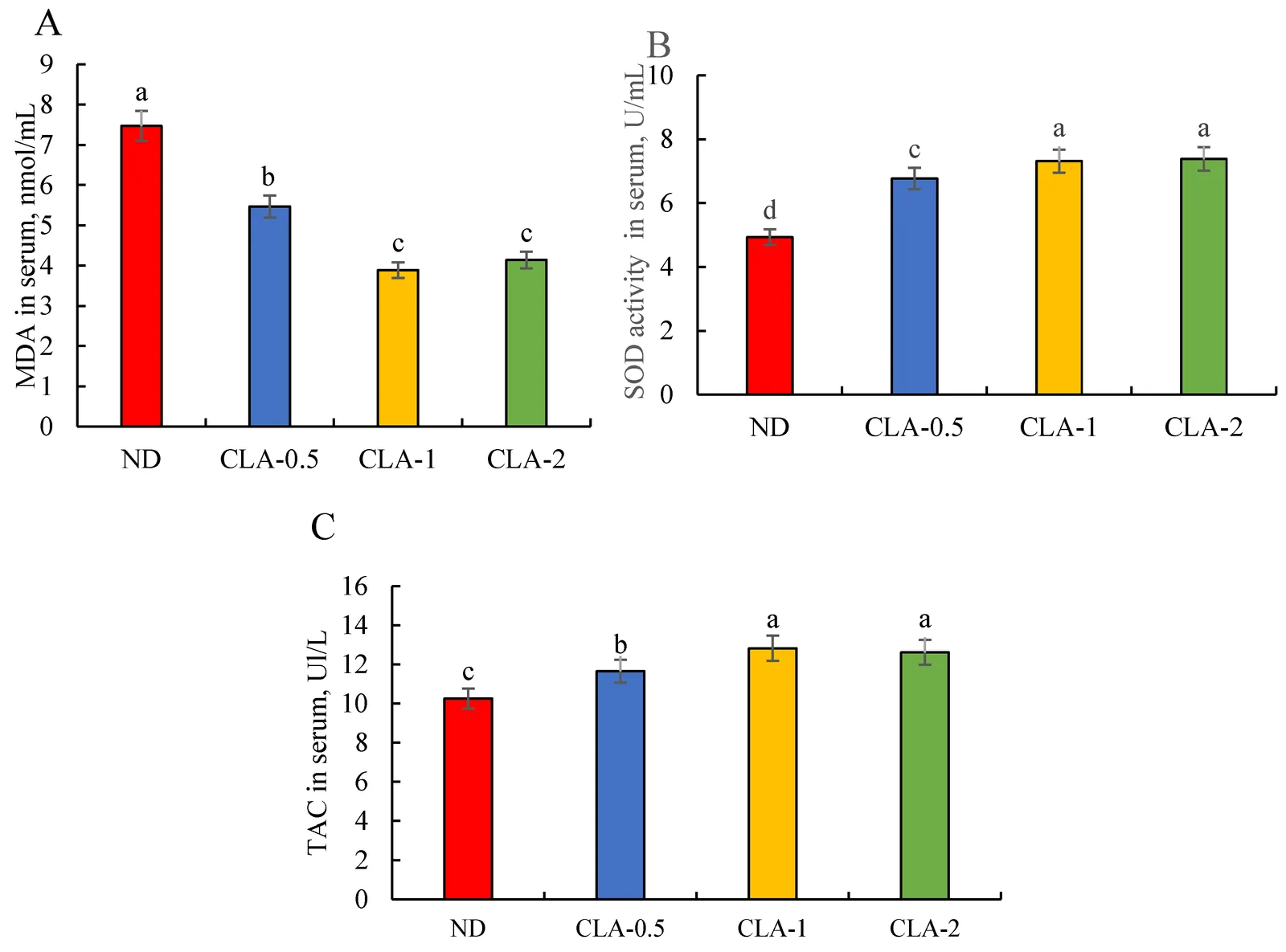
Serum levels of malondialdehyde (MDA) (**A**), superoxide dismutase (SOD) activity (**B**), and total antioxidant capacity (TAC) (**C**) in C57BL/6J mice fed diets containing different CLA concentrations. Serum samples were collected 24 h after LPS challenge. Values are presented as means ± SEM (*n* = 6 per group). Values with different lowercase letters (a–d) indicate significant differences at *p* < 0.05 according to Duncan’s multiple range test.

**Figure 5:**
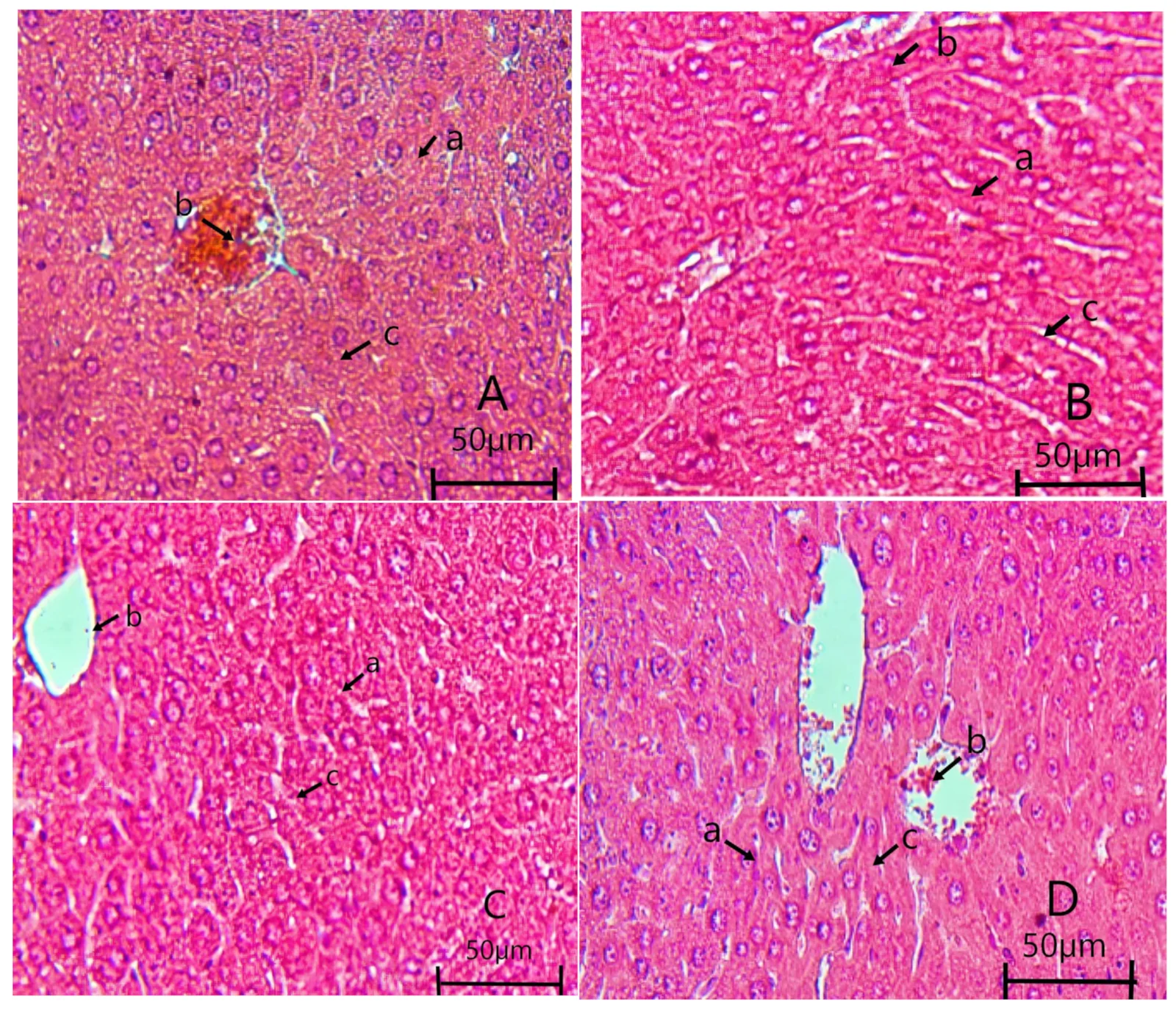
Histopathological images of mice liver sections, showing hepatocytes (a), inflammatory cells around the central vein (b), and hepatic sinusoidal cells (c). Panel (**A**) represents the ND group with eosinophilic cytoplasmic staining (cytoplasmic darkening) and obvious inflammatory infiltration. Panel (**B**) represents the CLA-0.5 group with some inflammatory infiltration. Panel (**C**) shows the CLA-1 group with less inflammatory infiltration. Panel (**D**) represents the CLA-2 group. Liver sections were obtained 24 h after LPS challenge. (Mag. 200×, Scale bar = 50 μm).

**Table 1:** Primer and annealing temperature list for qRT-PCR.

Gene	Accession Number	Forward Sequence (5′~3′)	Annealing Temperature (°C)
*GAPDH*	NM_001289726.2	F:TCAACGGGAAGCTCACTGG R:CCCCAGCATCGAAGGTAGA	60
*TNF-α*	NM-001278601.1	F:CGCTGAGGTCAATCTGC R: GGCTGGGTAGAGAATGGAA	58
*CXCL1*	NM_009909.3	F: GAGAGGGAATAGGG R: CTGCATTGGTTCAGTGGTT	60
*PPARγ*	XM-011245516.4	F: CACAATGCCATCAGGTTTGG R:GCTGGTCGATATCACTGGAGATC	59

## Data Availability

The raw data supporting the conclusions of this study are available from the corresponding author upon reasonable request.

## Supplementary Materials

The following supporting information can be downloaded at: https://www.mdpi.com/article/10.3390/biology15211992/s1, Figure S1: Schematic experimental timeline of dietary CLA intervention and LPS-triggered acute liver injury in C57BL/6J mice.
