# Supplementary material for: Dose-Dependent Effects of Conjugated Linoleic Acid on Hepatic Anti-Inflammatory Activity in Mice
Source: Biology (Basel). 2026 Nov 4;15(21):1992. doi: 10.3390/biology15211992 (PMC13761613; doi:10.3390/biology15211992)
Supplement: Supplementary file 1 [file biology-15-01992-s001.zip › biology-4594203-supplementary.pdf]

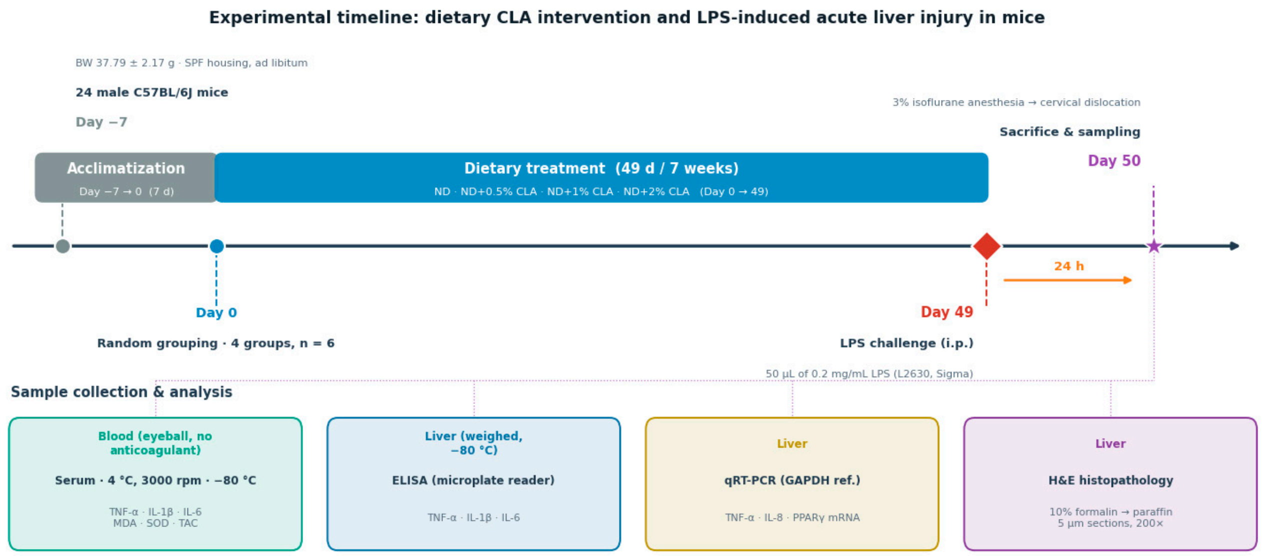

Figure S1：Schematic experimental timeline of dietary CLA intervention and LPS-triggered acute liver injury in C57BL/6J mice. Twenty-four male mice were subjected to a 7-day acclimatization period (Day −7 to Day 0). On Day 0, mice were randomly divided into four groups (n = 6 per group) and received 49 days of dietary intervention with normal diet (ND) or ND supplemented with 0.5%, 1%, or 2% CLA, respectively. On Day 49, all mice received an intraperitoneal injection of 50 μL LPS (0.2 mg/mL). Animals were sacrificed 24 h after LPS challenge (Day 50). Blood and liver tissues were harvested for serum biochemistry, ELISA, qRT-PCR and H&E histopathological analysis.
